# Compound from *Magnolia officinalis* Ameliorates White Matter Injury by Promoting Oligodendrocyte Maturation in Chronic Cerebral Ischemia Models

**DOI:** 10.1007/s12264-023-01068-z

**Published:** 2023-06-09

**Authors:** Zhi Zhang, Xin Shu, Qian Cao, Lushan Xu, Zibu Wang, Chenggang Li, Shengnan Xia, Pengfei Shao, Xinyu Bao, Liang Sun, Yuhao Xu, Yun Xu

**Affiliations:** 1 Department of Neurology, Nanjing Drum Tower Hospital Clinical College of Traditional Chinese and Western Medicine, Nanjing University of Chinese Medicine, Nanjing, 210008 China; 2 Department of Neurology, Nanjing Drum Tower Hospital, Affiliated Hospital of Medical School and State Key Laboratory of Pharmaceutical Biotechnology and Institute of Translational Medicine for Brain Critical Diseases, Nanjing University, Nanjing, 210008 China; 3 Jiangsu Key Laboratory for Molecular Medicine, Medical School of Nanjing University, Nanjing, 210008 China; 4 Jiangsu Provincial Key Discipline of Neurology, Nanjing, 210008 China; 5 Nanjing Neurology Medical Center, Nanjing, 210008 China

**Keywords:** Honokiol, White matter injury, Oligodendrocyte, Vascular dementia

## Abstract

Chronic cerebral hypoperfusion leads to white matter injury (WMI), which subsequently causes neurodegeneration and even cognitive impairment. However, due to the lack of treatment specifically for WMI, novel recognized and effective therapeutic strategies are urgently needed. In this study, we found that honokiol and magnolol, two compounds derived from *Magnolia officinalis*, significantly facilitated the differentiation of primary oligodendrocyte precursor cells (OPCs) into mature oligodendrocytes, with a more prominent effect of the former compound. Moreover, our results demonstrated that honokiol treatment improved myelin injury, induced mature oligodendrocyte protein expression, attenuated cognitive decline, promoted oligodendrocyte regeneration, and inhibited astrocytic activation in the bilateral carotid artery stenosis model. Mechanistically, honokiol increased the phosphorylation of serine/threonine kinase (Akt) and mammalian target of rapamycin (mTOR) by activating cannabinoid receptor 1 during OPC differentiation. Collectively, our study indicates that honokiol might serve as a potential treatment for WMI in chronic cerebral ischemia.

**Supplementary Information:**

The online version contains supplementary material available at 10.1007/s12264-023-01068-z.

## Introduction

Chronic cerebral hypoperfusion is a pathophysiological factor contributing to white matter injury (WMI) [1, 2], which displays punctate, patchy, or confluent T2-weighted or FLAIR (fluid attenuated inversion recovery) hyperintensity on magnetic resonance imaging (MRI) and mainly involves the periventricular region [3, 4]. More evidence indicates that white matter plays a vital role in the coordination of various cerebral regions. Severe WMI can cause cognitive impairment and even lead to vascular dementia, resulting in a large social and economic burden [5]. However, apart from controlling high-risk factors of WMI, there is still a lack of widely accepted and effective therapeutic strategies [6].

The white matter consists of axons extending from neuronal somata and myelin sheaths which are highly sensitive to hypoxic-ischemic injury [7, 8]. The myelin sheath not only enables rapid neuronal impulse conduction but also provides metabolic support for axons [9, 10]. Oligodendrocytes (OLs) are the myelin-generating cells in the central nervous system (CNS). Once damaged, they can be replenished through the proliferation and differentiation of oligodendrocyte precursor cells (OPCs) [11]. Notably, WMI and cognitive impairment associated with chronic cerebral ischemia are alleviated by promoting OL maturation, which provides a promising target for the treatment of WMI after chronic cerebral ischemia [12–14]. Furthermore, the progression and maturation of OL lineage cells are under tight transcriptional and post-transcriptional control. For example, the expression of neuron-glial antigen 2 (NG2) is down-regulated in OPCs after differentiating into mature OLs, while the expression of Sox10 is up-regulated during OPC differentiation [15]. Besides, other transcription factors, such as myelin regulatory factor (MYRF) and Nkx2.2, are also critical for OL maturation [16, 17]. All of these provide many identified molecules and therapeutic targets for research on OL maturation.

Honokiol and magnolol are two isomers of the main active ingredients in *Magnolia officinalis*; they are widely used in the treatment of cancers, neurological disorders, gastrointestinal diseases, and other disorders [18–22]. Both of them can permeate the blood-brain barrier (BBB) and provide broad protection in various neurological and psychiatric diseases [9, 23–26]. Recent research indicates that honokiol or magnolol can modulate the phosphoinositide 3-kinase (PI3K)/Akt/mTOR pathway and the extracellular signal-regulated kinase (ERK) pathway [27, 28]. These pathways may be associated with OPC differentiation and remyelination [11, 12]. However, it remains unclear whether honokiol or magnolol can alleviate WMI and promote remyelination after chronic cerebral ischemia.

In this study, we used the bilateral carotid artery stenosis (BCAS) mouse model to induce WMI and cognitive impairment associated with chronic cerebral hypoperfusion [29–31]. We found that both honokiol and magnolol could promote OPC differentiation without affecting OPC survival and proliferation. Furthermore, honokiol also ameliorated WMI, improved cognitive decline, and promoted OPC differentiation in BCAS mice through the CB1/Akt/mTOR pathway. In summary, these data indicated that honokiol might serve as a potential therapeutic drug to relieve WMI of chronic cerebral hypoperfusion and ameliorate the associated cognitive decline.

## Materials and Methods

### Cell Culture and Treatment

OPCs were prepared from newborn C57BL/6 pups (postnatal 0–2; P0–2). After removing the meninges, the cortical tissues were mechanically minced and dissociated. Then, the tissue suspension was filtered through a 70-μm nylon cell strainer and plated on poly-D-lysine (Sigma, St. Louis, USA)-coated culture plates or dishes with Dulbecco’s modified Eagle’s medium/F12, B27, penicillin/streptomycin (Gibco, CA, USA) proliferation medium containing 15 ng/mL platelet-derived growth factor-AA (GenScript, Beijing, China) and 5 ng/mL basic fibroblast growth factor (GenScript) for 7–10 days before differentiation. Triiodothyronine (T3; R&D Systems, MN, USA) and ciliary neurotrophic factor (CNTF; GenScript) were used to induce the differentiation of OPCs. Honokiol (MCE, Shanghai, China) and magnolol (MCE) were dissolved in DMSO (Sigma, USA) and added to primary OPCs for further tests.

### Cell Viability Assessment

After treatment with various concentrations of honokiol or magnolol (5, 10, 20, 50, and 100 μmol/L) for 24 h or 72 h, cell counting kit-8 (CCK-8) (Dojindo Laboratories, Kumamoto, Japan) analysis was used to measure the viability of OPCs cultured in 96-well plates. Briefly, 10 μL of CCK-8 solution was added to each well. After incubation at 37°C for 2 h, the absorbance at 450 nm was measured using a microplate reader (Tecan, Zurich, Switzerland).

### TdT-mediated dUTP Nick End Labeling (TUNEL) Staining

After treatment with honokiol and magnolol (5 and 10 μmol/L) in the proliferating medium for 72 h, the primary OPCs were fixed in 4% formaldehyde for 20 min. Quantification of DNA fragmentation was measured using an *in situ* apoptosis detection kit according to the manufacturer’s protocol (Abbkine, Suzhou, China). The TUNEL-positive cells were observed using a confocal microscope (FV3000, Olympus, Tokyo, Japan).

### Animals and Bilateral Common Carotid Artery Stenosis

Groups of 8-week-old male C57BL/6 mice were purchased from the Model Animal Research Center, Nanjing University. These mice were housed at 22–25°C and fed standard food and water based on institutional guidelines. All procedures were approved by the Animal Care Committee of Nanjing University (2021AE01068).

The BCAS model was established as described previously [32]. Briefly, mice were first anesthetized with Avertin (2.5%, Sigma). The common carotid arteries were exposed through a middle cervical incision. The operation was performed using micro-coils (inner diameter 0.18 mm, pitch 0.50 mm, total length 2.5 mm, Sawane Spring Co., Hamamatsu, Japan) specifically designed for the mouse to induce chronic cerebral hypoperfusion. Sham-operated mice underwent all procedures except for micro-coil implantation. Body temperature was maintained by a heating pad after the surgery. Mice in the honokiol group were intraperitoneally injected with 10 mg/kg honokiol (pre-dissolved in DMSO) in 200 μL saline each day for 30 days from BCAS 1 month, and mice in the sham group and the control group were injected daily with equal DMSO in 200 µL saline at the same time.

### Immunofluorescence Analysis

The mice were deeply anesthetized by 2.5% Avertin (Sigma) and perfused with ice-cold 0.1 mol/L phosphate-buffered saline (PBS), followed by 4% formaldehyde in 0.1 mol/L PBS. The cerebrums were removed from the cranial fossae and immersed in 15% and 25% sucrose at 4°C for 24 h in each concentration. Then, those brains were cut at 20 μm on a rotary microtome (Thermo Fisher, Waltham, USA). The sections were permeabilized with 0.25% Triton X-100, blocked with 2% bovine serum albumin, and then incubated with anti-Ki67, anti-NG2, anti-MBP, anti-Olig2, anti-CC1, anti-GFAP, or anti-Iba1 overnight at 4°C. After 3 washes, those sections were incubated with secondary antibodies (Invitrogen, Carlsbad, USA) for 2 h at room temperature. Finally, antifade mounting medium with DAPI (Beyotime, Shanghai, China) was added to each section to decrease the fluorescence quenching. The primary OPCs were fixed in 4% formaldehyde for 15 min and followed a similar protocol. The laser scanning confocal microscope (FV3000, Olympus) was used to obtain fluorescence images. The images were further analyzed by ImageJ v1.8 software (NIH, MD, USA).

### Black Gold II Staining

Black-Gold II is a haloaurophosphate complex that is able to identify myelin within the central nervous system. Black-Gold II was dissolved in 0.9% saline to prepare 0.3% Black-Gold II solution (Biosensis, CA, USA). After incubating frozen brain sections in 65°C Black-gold II solution for 10 min until the finest myelinated fibers turned dark red and the degree of myelin impregnation was verified under the microscope. After washing with distilled water, the sections were immersed in 1% sodium thiosulfate for 3 min at 65°C for fixation. Subsequently, the sections were washed with distilled water, dehydrated in ethanol, and cleared in xylene for 2 min. The sections were coverslipped and analyzed under an inverted microscope (Olympus IX73).

### Western Blotting

Protein samples were collected from the corpus callosum (CC) of mice and primary OL lineage cells. Tissue and cell samples were segregated and homogenized with RIPA (Thermo Fisher, USA) and protease inhibitor (Thermo Fisher). The cellular debris was removed by centrifugation at 4°C, 13,000 r/min for 30 min, and the supernatant was diluted to the same concentration and mixed with 1× loading buffer. For each sample, >20 μg of protein was subjected to SDS-PAGE and then transferred to polyvinylidene difluoride membranes (Millipore, MA, USA). After blocking in 5% skim milk for 2 h at room temperature, the membranes were incubated overnight at 4°C with primary antibodies against NG2, MAG, PLP1, MBP, Ki67, eNOS, Sox10, mTOR, p-mTOR, p-Stat3, Stat3, Akt, or p-Akt at 1:1000 dilution, and GFAP, Erk1/2, or p-Erk1/2 at 1:2000 dilution using 5% milk in TBST (0.1% Tween-20) overnight on a shaker at 4°C. Anti-GAPDH antibody was used as loading controls. After incubation with primary antibodies, the membranes were washed three times in TBST, and then separately incubated with the corresponding secondary antibodies. Immunoreactivity was visualized with a Western blotting chemiluminescent substrate (Millipore).

### Behavioral Tests

The mice performed the following behavioral tests 2 months after BCAS.

#### Open-field Test (OFT)

The OFT was designed to evaluate autonomous exploratory behavior and the mental tension of the mice when facing a relatively new environment. The mice were placed in a 40 cm × 40 cm × 40 cm box and allowed to explore freely for 10 min. The bottom of the box was separated into 4 × 4 grids. The total distance the mice had traveled and the time spent in the corner and central grids were recorded and analyzed using Anymaze software (Stoelting, Chicago, USA).

#### Novel Object Recognition (NOR)

The NOR test is commonly used to investigate short-term learning and working memory. The mouse was placed in a 20 cm × 20 cm × 30 cm box without any objects and left for 15 min on three consecutive days during the adaptive trials. During the training process, the mice were allowed to explore 2 identical objects for 10 min, and 1.5 h later, were placed in the same box in which one of the training objects was replaced with a novel one. The total exploration time for both objects was recorded and analyzed.

#### Fear Conditioning (FC)

The mice were placed in a conditioning chamber (Panlab, Barcelona, Spain) for 5 min without any stimulation and subsequently received a tone-stimulus (30 s, 80 dB) before one foot shock (2 s, 0.75 mA). The mice were allowed to stay in the chamber for 1 min after the shock to evaluate post-shock freezing. Context-dependent memory was assessed 1 day after training. The mice were allowed to freely explore the same chamber for 5 min without any stimulation. However, cue-dependent memory was also examined in a novel chamber. The mice were allowed to freely explore the novel chamber for 1 min without any stimulation and subsequently 4 min with tone stimuli. The chamber was cleaned with 75% alcohol after each test, and the freezing time was determined using Packwin software (Panlab).

#### Y Maze Test

The Y maze test is a highly effective way to assess the condition of short-term memory and working memory. The Y maze consists of 3 arms installed in the “Y” shape. Each arm was 40 cm × 8 cm × 10 cm in size. During training, the mice were placed in the center of the maze while one arm was sealed by an opaque board. When the test began, all three arms were opened and the mice were allowed to freely explore for 8 min. The time which each mouse spent in each arm was recorded, and the time spent in the target arm (new and opened arm) was analyzed by GraphPad Prism 7.0 (GraphPad, CA, USA).

### Transmission Electron Microscopy (TEM)

After the mice of all groups were euthanized, 1 mm × 1 mm × 1 mm pieces of tissue from the CC were taken, and the following processing procedure was applied as in the previously reported protocol [32]. In brief, the pieces of tissue were incubated in 2.5% glutaraldehyde in PBS at 4°C overnight and then fixed in 2% osmium tetroxide in PBS for 2 h. After alcohol gradient dehydration, the samples were embedded in epoxy resin. Subsequently, semi-thin sections that were cut on an ultramicrotome were stained with 1% toluidine blue, uranyl acetate, and lead citrate. Then, images were screened by TEM (HT7800, Hitachi, Tokyo, Japan). The G-ratio (diameter of the axon/diameter of the myelinated fiber) and thickness [diameter of the myelinated fiber − diameter of the axon)/2] of myelin sheaths per animal were calculated and analyzed by ImageJ.

### EdU Staining

EdU (5-ethynyl-2’-deoxyuridine) staining was used to identify newly-generated cells. The EdU (Invitrogen) was delivered to each mouse weekly at 10 mg/kg by intraperitoneal injection from 6 weeks to 8 weeks after BCAS. The mice were sacrificed 2 months after BCAS and the brain was cut into frozen slices. Then the slices were fixed in 4% paraformaldehyde in PBS for 15 min. Next, the slices were washed twice with 3% BSA and permeabilized with 0.5% Triton X-100 in PBS for 20 min. The slices were again washed twice with 3% BSA in PBS and then incubated with a cocktail containing Click-iT™ reaction buffer, CuSO_4_, Alexa Fluor^®^ 647 Azide, and reaction buffer additive (Invitrogen) for 30 min. The slices were then washed twice with PBS and further immunofluorescence staining was applied before using a laser scanning confocal microscope (FV3000, Olympus). All steps were carried out at room temperature.

### Laser Speckle Contrast Imaging

The condition of cerebral blood perfusion was measured using a High-resolution Laser Speckle Contrast Imager (PeriCam PSI System, Perimed AB, Jakobsberg, Sweden). Two months after BCAS, the mice were anesthetized with avertin (2.5%, Sigma) and shaved. The skull was exposed by a midline incision. The ROI (region of interest) was set between bregma and lambda to measure the hemisphere perfusion on both sides. Perfusion images of CBF (cerebral blood flow) were captured using the PSI system with a camera installed 10 cm above the skull. Captured images were analyzed using the customized PIMSoft program (Perimed AB).

### Statistical Analysis

The data are presented as the mean ± SD and were analyzed using GraphPad Prism 7.0. Statistical differences were determined by one-way analysis of variance (ANOVA) followed by *post hoc* multiple-comparisons tests (Bonferroni’s correction) to analyze differences among three or more groups with one independent variable. A *P-*value <0.05 was considered statistically significant.

## Results

### Effects of Honokiol and Magnolol on OPC Survival and Proliferation

**Fig. 1:**
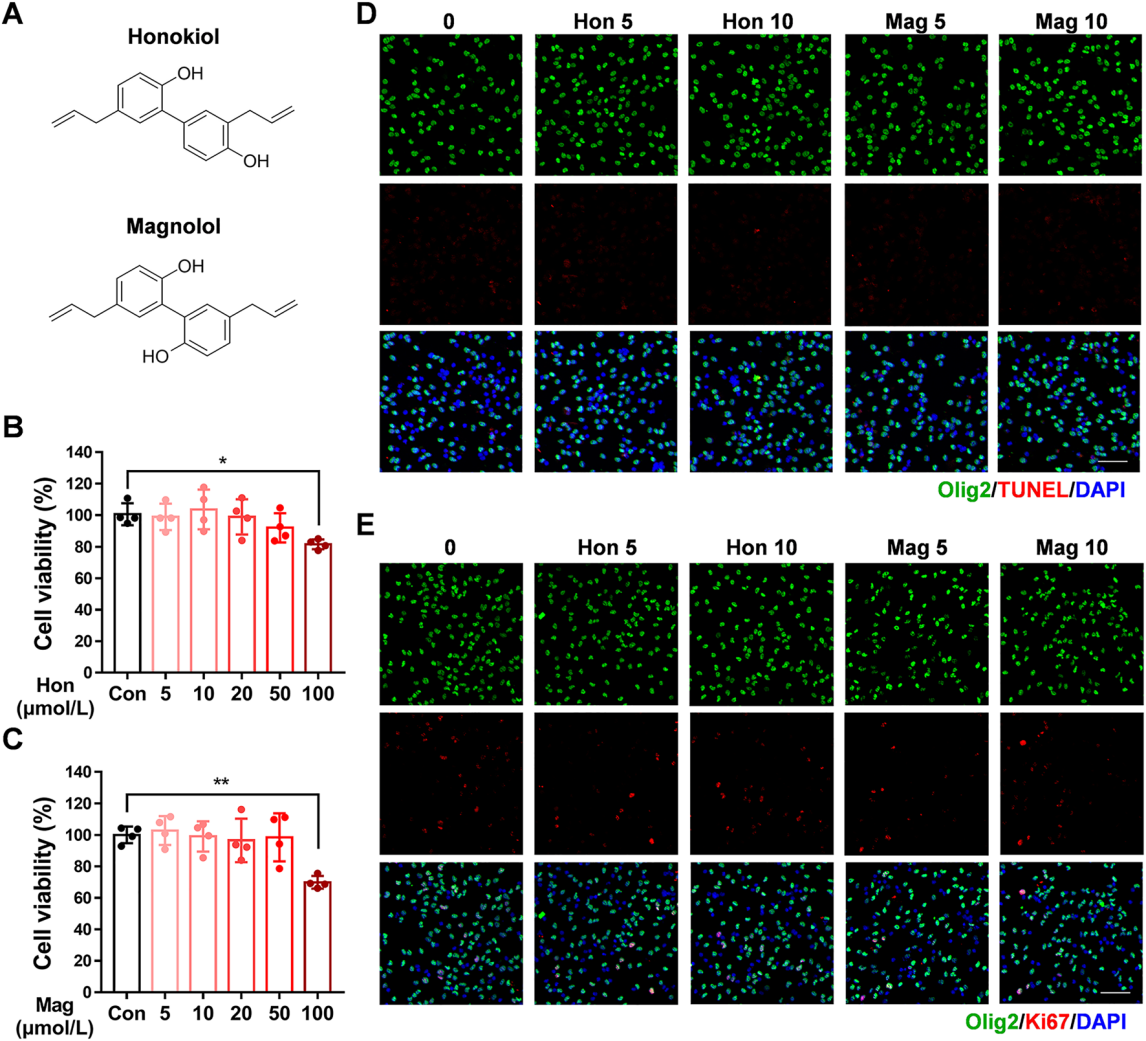
Effects of honokiol and magnolol on OPC survival and proliferation. **A** Chemical structures of the isomeric polyphenolic components derived from *Magnolia officinalis*. **B, C** Viability of OPCs after treatment with honokiol (Hon; 5–100 μmol/L) (**B**) or magnolol (Mag; 5–100 μmol/L) (**C**) for 24 h measured by the CCK-8 test, *versus* the control group. The results are presented as the mean ± SD. *n* = 4, *P <0.05, **P <0.01, one-way ANOVA. **D, E** After primary OPCs were treated with 5 or 10 μmol/L honokiol and magnolol in proliferating medium for 72 h, OPCs were labeled by TUNEL staining (red) (**D**) or immunofluorescence staining of Ki67 (red) (**E**) and Olig2 (green). DAPI stained nuclei. Scale bars, 50 μm.

We first investigated the cytotoxicity of honokiol and magnolol, two isomeric polyphenolic components derived from *Magnolia officinalis* (Fig. 1A), mixing them with primary OPCs for 24 h in the CCK-8 test. Our results demonstrated that treatment with neither honokiol (5–50 μmol/L) nor magnolol (5–50 μmol/L) affected cell viability (Fig. 1B, C). However, when the concentration reached 100 μmol/L, the viability of the OL lineage cells decreased significantly (Fig. 1B, C). Thus, we chose concentrations <50 μmol/L for further research. Moreover, the results of TUNEL staining, Ki67 immunofluorescence, and the CCK-8 test for 72 h demonstrated that both 5 or 10 μmol/L honokiol and magnolol had no effect on OPCs survival or proliferation (Figs 1D, E and S1A, B).

### Honokiol and Magnolol Promote OL Maturation *In Vitro*

**Fig. 2:**
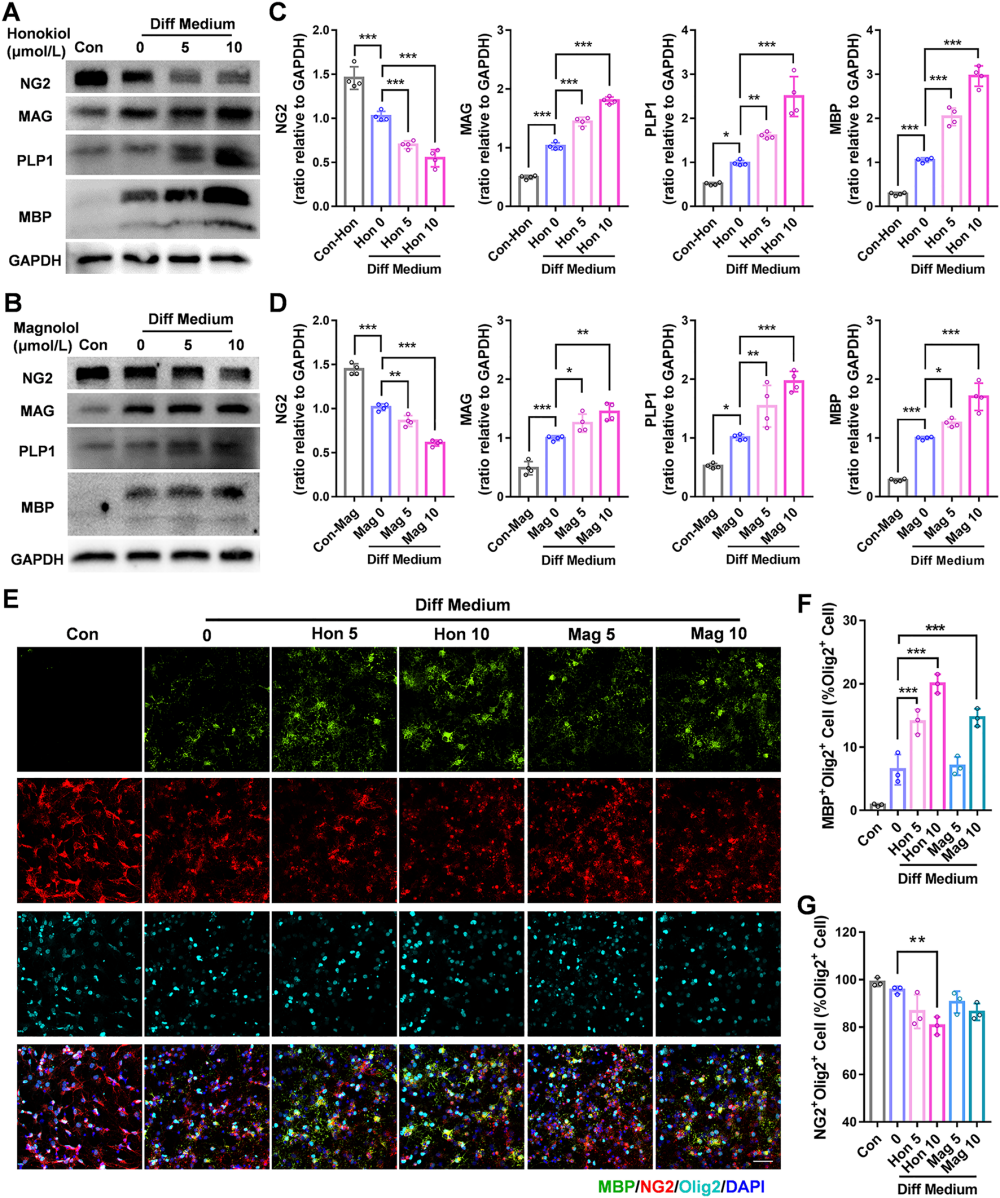
Honokiol and magnolol promote OL maturation *in vitro*.** A** Western blots of expression of the myelin proteins MBP, MAG, PLP1, and the OPC marker NG2 in primary OPC treated with 5 or 10 μmol/L honokiol in differentiating medium for 24 h. **B** Western blots of expression of the myelin proteins MBP, MAG, PLP1, and the OPC marker NG2 with GAPDH as a loading control in OPCs treated with 5 or 10 μmol/L magnolol in differentiating medium for 24 h. **C, D** Analysis of Western blots as in** A** and **B**. The results are presented as the mean ± SD. *n* = 4, *P <0.05, **P <0.01, ***P <0.001, one-way ANOVA. **E** by immunofluorescence staining of MBP (green), NG2 (red), and Olig2 (cyan) in OPCs and OLs after primary OPCs were treated with 5 or 10 μmol/L honokiol and magnolol in differentiating medium for 24 h. Nuclei are stained with DAPI. Scale bar, 50 μm. **F** Percentages of MBP^+^ Olig2^+^ cells in Olig2^+^ cells. **G** Percentages of NG2^+^ Olig2^+^ cells in Olig2^+^ cells. The results are presented as the mean ± SD. *n* = 3, **P <0.01, ***P <0.001, one-way ANOVA. Con, control; Hon, honokiol; Mag, magnolol; Diff, different.

Then, we examined the effects of the two components on OL maturation and OPC differentiation *via* Western blotting. At 7 days *in vitro*, 5 and 10 μmol/L of honokiol and magnolol were separately added to OPC differentiating medium (containing 15 μmol/L T3 and 10 ng/mL CNTF). The results showed that both honokiol and magnolol raised the expression of mature OL myelin proteins (MBP, PLP1, and MAG) after 48 h, as well as decreasing the expression of NG2, an OPC marker, compared with the control group (Fig. 2A–D). Moreover, the effect of honokiol was more prominent than that of magnolol. To determine the proportion of OPCs that differentiate into mature OLs after drug treatments, OLs and OPCs were labeled by immunofluorescence staining for MBP and NG2 respectively, while both were labeled by Olig2. Similarly, honokiol and magnolol treatment significantly increased the proportion of OLs in all OL lineage cells, and honokiol displayed greater effects than magnolol (Fig. 2E–G). Meanwhile, we found that only 10 μmol/L honokiol slightly evaluated certain mature OL proteins of OL lineage cells in proliferating medium (without T3) for 72 h (Fig. S1B–G). These results thus suggested that honokiol might be a better enhancer of the T3-potentiated differentiation of OPCs than magnolol. Thus, honokiol was chosen for further *in vivo* and *in vitro* experiments.

### Honokiol Attenuates Vascular Cognitive Decline and Myelin Injury in a Rodent Model

**Fig. 3:**
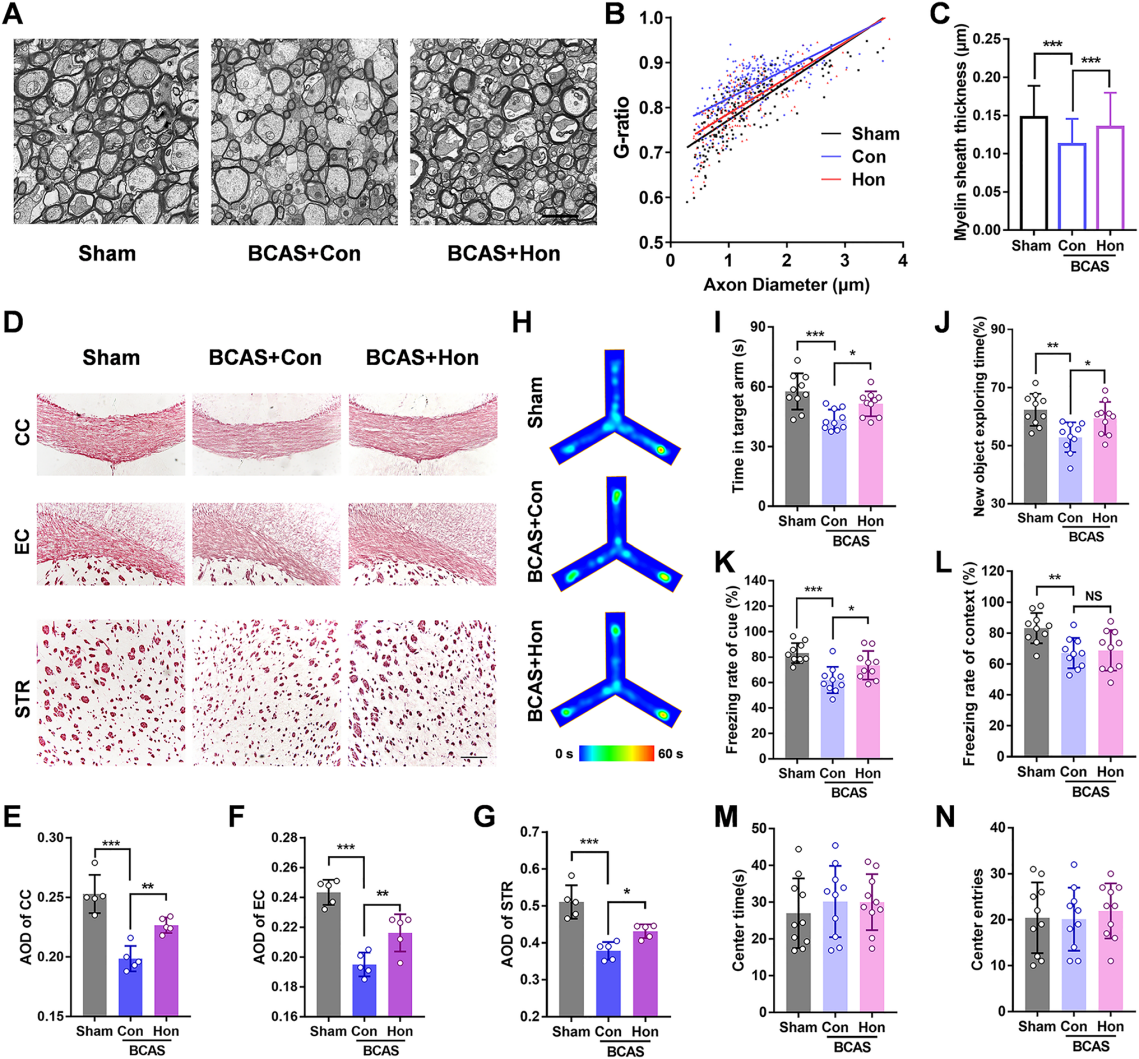
Honokiol attenuates vascular cognitive decline and myelin injury in the BCAS model. **A** Representative electron microscopic images of the corpus callosum at BCAS 2 months. Scale bar, 2 μm. **B** G-ratios (diameter of the axon/the diameter of the myelinated fiber) (equal slope: *P-*value = 0.0077). **C** Myelin sheath thickness [(diameter of the myelinated fiber − diameter of the axon)/2]. All results are expressed as the mean ± SD. *n* = 3 mice per group, *n* >100 axons per group, ***P <0.001. one-way ANOVA. **D** Black-Gold staining for the integrity of myelin in the corpus callosum (CC), external capsule (EC), and striatum (STR) in each group after BCAS 2 months. Scale bar, 250 μm. **E–G** Average optical density (AOD) of Black-Gold staining in the corpus callosum (**E**), external capsule (**F**), and striatum (**G**). All results are expressed as the mean ± SD. *n* = 5, *P <0.05, **P <0.01, ***P <0.001. one-way ANOVA. **H** Heat maps showing the average time in the target arm for each group in the Y maze test. **I** Time in the target arm in the Y maze test. **J** Exploration time of the new object in the NOR test. **K** Freezing rate in the cued FC test. **L** Freezing rate in the contextual FC test. **M, N** Analysis of the OFT, including center time (**M**) and center entries (**N**) which reflect mobility or psychiatric conditions of mice. All results are expressed as the mean ± SD. *n* = 10, *P <0.05, **P <0.01, ***P <0.001, NS no statistically significant difference, one-way ANOVA.

Then we considered that honokiol could also improve dysfunctional OLs *in vivo*, so BCAS, a well-established model of chronic cerebral ischemia was used to investigate the effect of honokiol on WMI and vascular cognitive impairment. One month after the BCAS, the mice were intraperitoneally injected with 20 mg/kg/day honokiol for 30 days. First, the CC was examined by electron microscopy 2 months after BCAS. The data showed a decreased G-ratio [95% confidence intervals of slopes: Sham = 0.07665 to 0.09364, Con (control) = 0.05624 to 0.07416, Hon (honokiol) = 0.06809 to 0.09055] and an increased myelin sheath thickness in the BCAS + Hon group relative to the BCAS + Con group (Fig. 2A–C). These results indicated that honokiol treatment improved the WMI in BCAS mice. Then, we assessed the integrity of myelin by Black-Gold staining. The integrity of the myelin sheath in the CC, external capsule (EC), and striatum (STR) after BCAS was compromised but significantly improved after treatment with honokiol (Fig. 3D–G). In addition, behavioral analysis of mice at 2 months after the BCAS showed significant deficiencies in the Y-maze test, NOR test, cued fear conditioning (FC) test, and contextual FC test. Moreover, the honokiol treatment group had better behavioral performance in the Y-maze test, NOR test, and cued FC test (but not the contextual FC test) than the vehicle group (Fig. 3H–L), indicating that honokiol could improve the working memory and tone-dependent fear memory rather than context-dependent fear memory caused by chronic cerebral ischemia. In order to exclude the interference of locomotor ability, an open field test was applied. The center time and center entries showed no difference among all groups, suggesting that the defects in cognition-related tests were caused by cognitive impairment but not mobility or psychiatric conditions (Fig. 3M, N). Taken together, the above results demonstrated that honokiol could attenuate myelin injury after chronic cerebral ischemia and improve vascular cognitive decline.

### Honokiol Promotes OL Maturation and OPC Differentiation in the BCAS Model

**Fig. 4:**
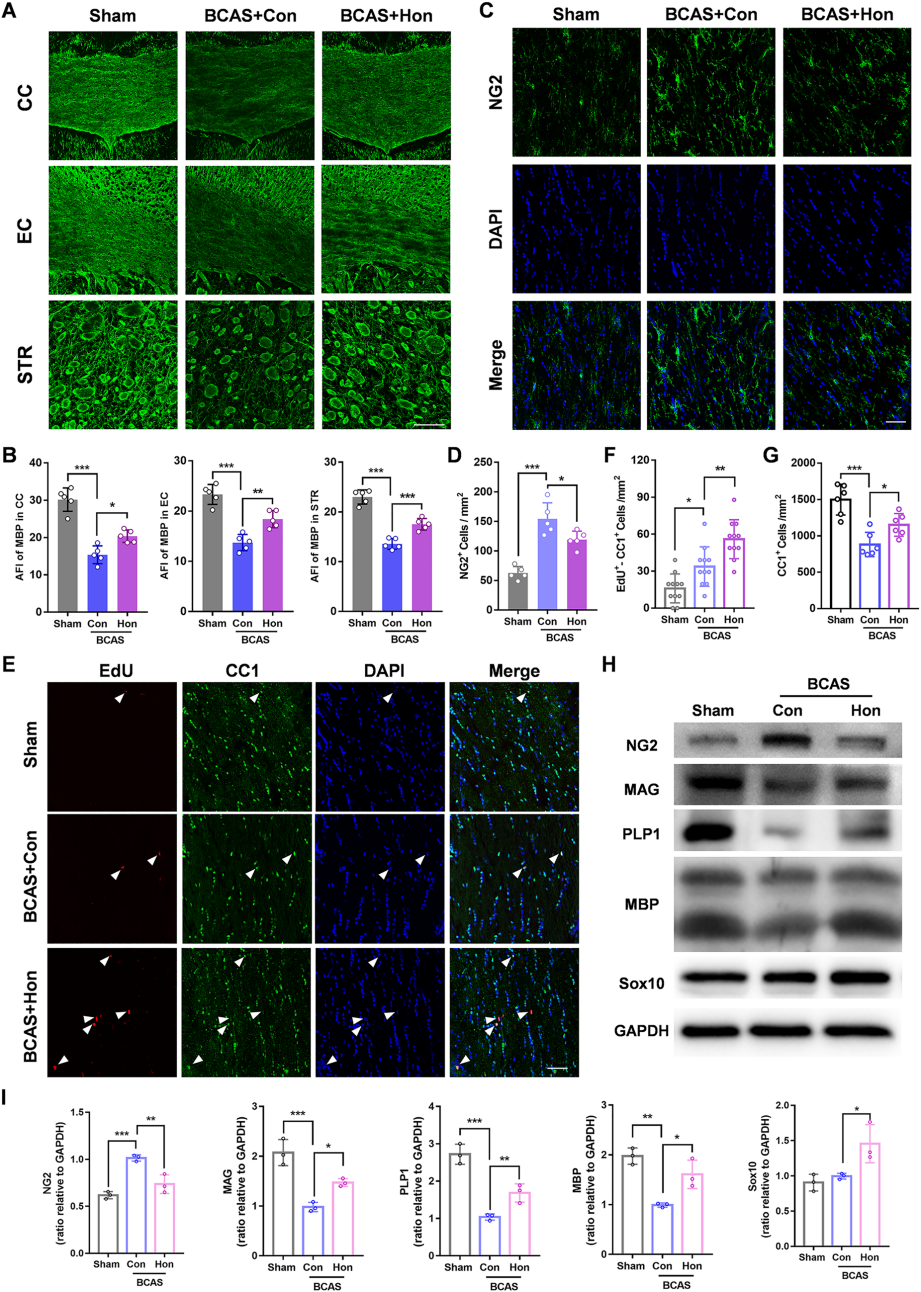
Honokiol promotes OL maturation and OPC differentiation in the BCAS model. **A** Immunofluorescence staining of MBP in the corpus callosum (CC), external capsule (EC), and striatum (STR) for each group 2 months after BCAS. Scale bar, 50 μm. **B** Average fluorescence intensity (AFI) of MBP staining in the CC, EC, and STR. **C** Immunofluorescence staining of NG2 to label OPCs in the CC 2 months after BCAS. Nuclei are stained with DAPI. Scale bar, 50 μm. **D** Numbers of NG2^+^ cells per mm^2^ as in **C**. **E** Newborn cells are marked by EdU (red) staining and nuclei of mature OLs are labeled by CC1 (green) and DAPI. The EdU^+^ and CC1^+^ cells were recorded in the CC for each group. Scale bar, 50 μm. White arrowheads indicate EdU^+^CC1^+^ cells. **F, G** Numbers of EdU^+^CC1^+^ cells and total CC1^+^ cells per mm^2^ as in **E**. **H** Western blots of the expression of NG2, MBP, MAG, PLP1, and Sox10 in the CC with GAPDH as a loading control. **I** Quantitative analysis of Western blots as in **H**. The results are presented as the mean ± SD. *n* = 3, *P <0.05, **P <0.01, ***P <0.001, one-way ANOVA.

To test whether honokiol can affect OL maturation and OPC differentiation *in vivo*, we first tested the MBP expression by immunofluorescent staining and found that honokiol alleviated the loss of MBP compared to the control group after BCAS (Fig. 4A, B). Meanwhile, honokiol raised the level of the mature OL proteins MBP, MAG, and PLP1 after BCAS (Fig. 4H, I). Then we measured the expression of an OPC marker, NG2, by immunofluorescent staining and Western blot in the white matter region. Remarkably, the expression of NG2 protein and the number of NG2-positive cells in the corpus callosum were increased after BCAS. However, honokiol treatment showed less elevation than the control group, which indicated that OPC compensation increased due to myelin injury after BCAS, and honokiol might promote more OPC differentiation (Fig. 4C, D, H, I). Moreover, we found no significant change in Ki67^+^NG2^+^ cells around the subventricular zone (SVZ) after honokiol treatment, indicating that honokiol had no effect on OPC proliferation *in vivo* (Fig. S2A–C). 5-Ethynyl-2'-deoxyuridine (EdU) is a reagent widely used to label newborn cells *in vivo,* and CC1 was used as a marker of mature OLs. Newborn OLs (EdU^+^/CC1^+^ cells) and total OLs (CC1^+^ cells) in the corpus callosum were significantly increased in the honokiol-treated group, confirming the hypothesis that honokiol can ameliorate WMI by promoting OL maturation (Fig. 4E–G). In addition, we found that Sox10, an important transcriptional factor inducing OPC differentiation, was also up-regulated after honokiol treatment (Fig. 4H, I). Similarly, 5 or 10 μmol/L honokiol could also up-regulate Sox10 in primary OPCs *in vitro* (Fig. 6A). These data indicated that honokiol promoted OL maturation and OPC differentiation in BCAS mice.

### Honokiol Barely Affects Cerebral Blood Flow While Inhibiting the Activation of Astrocytes Rather than Microglia *In Vivo*

**Fig. 5:**
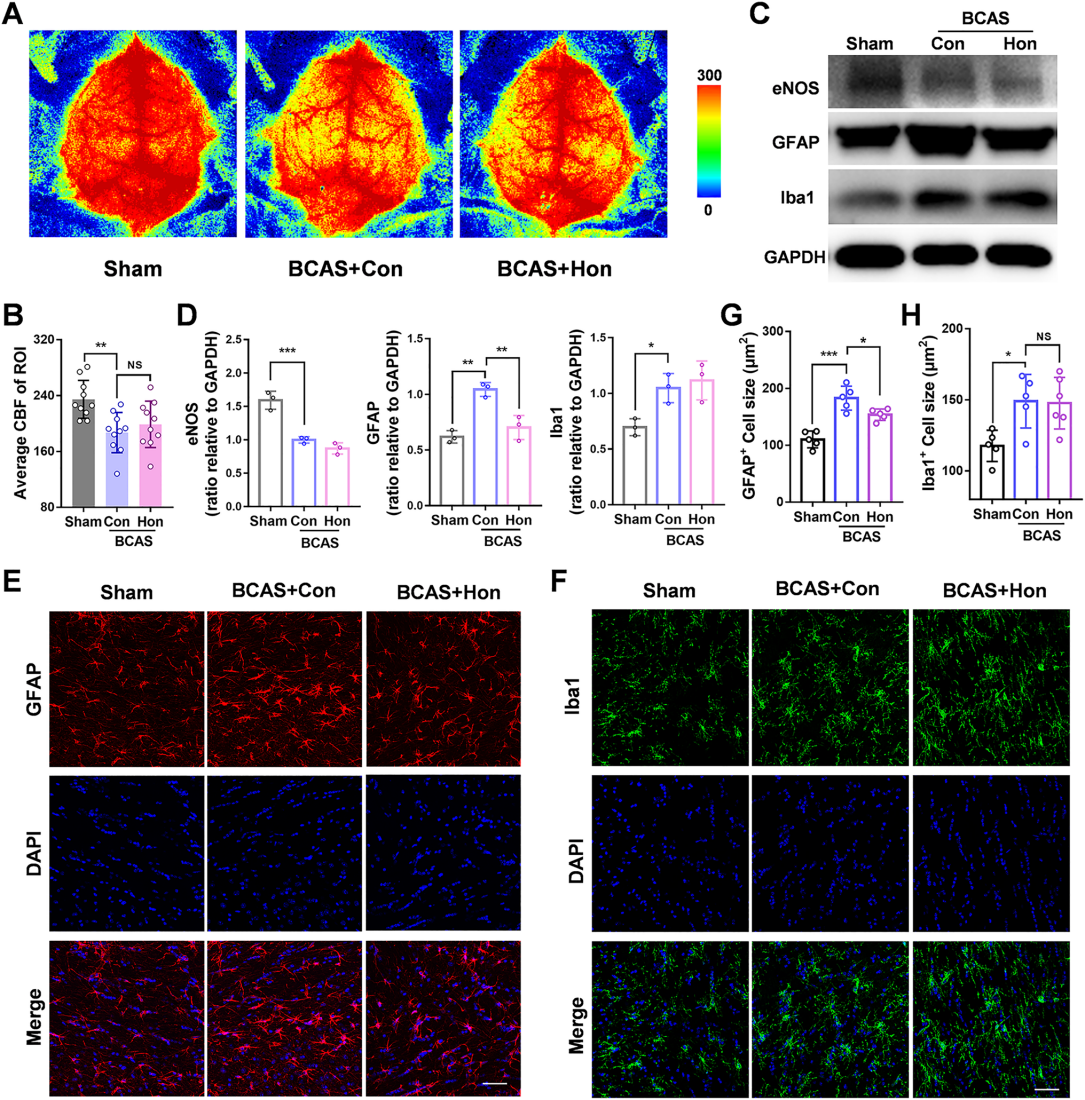
Effect of honokiol on cerebral blood flow (CBF) and activation of astrocytes and microglia after BCAS.** A** Representative CBF images for each group measured by laser speckle contrast imaging at 2 months after BCAS. **B** Average CBF of the ROI as in **A**. **C** Western blots of the expression of eNOS, GFAP, and Iba1 in the corpus callosum with GAPDH as a loading control. **D** Analysis of the Western blots as in** C**. All results are expressed as the mean ± SD. *n* = 3, *P <0.05, **P <0.01, ***P <0.001, NS no statistically significant difference, one-way ANOVA. **E, F** Immunofluorescence staining of GFAP (green) (**E**) and Iba1(red) (**F**) in the corpus callosum at 2 months after BCAS. Nuclei are stained with DAPI. Scale bar, 50 μm. **G** The average size (μm^2^) of GFAP^+^ cells as in** E**. **H** The average size (μm^2^) of Iba1^+^ cells as in** F**. All results are expressed as the mean ± SD. *n* = 5, *P <0.05, **P <0.01, ***P <0.001, NS no statistically significant difference, one-way ANOVA.

Since the main pathological change in the BCAS model is the reduction of CBF [33], we explored whether honokiol affected CBF by using laser speckle contrast imaging. Honokiol had no effect on CBF compared to the control group, while CBF was significantly reduced in the ROI 2 months after BCAS (Fig. 5A, B). We also demonstrated that eNOS (endothelial nitric oxide synthase), a vasodilator released from endothelial cells, decreased significantly in BCAS mice, but was not changed after honokiol treatment (Fig. 5C, D). However, honokiol affected neither CBF nor cerebral vasodilation after chronic cerebral ischemia.

Recent research has reported that honokiol can reduce the inflammatory response of primary microglia and astrocytes [24, 34]. Since neuroinflammation has also been reported to aggravate WMI [35–37], we also assessed the effect of honokiol on glial cells in the BCAS model. We used Western blot and immunofluorescent staining to analyze the reactive astrocyte marker GFAP (glial fibrillary acidic protein) and the microglia marker Iba1 (ionized calcium-binding adaptor molecule 1) which is up-regulated in activated microglia. Honokiol also reduced the expression of GFAP and inhibited the activation of astrocytes after BCAS (Fig. 5C–E, G), but did not affect Iba1 expression and microglial activation (Fig. 5C, D, F, H). Besides, we found that galectin-3, an inflammatory marker [38, 39], in microglia was slightly up-regulated 2 months after BCAS, and was not significantly changed after honokiol treatment (Fig. S2D–F). Therefore, our results showed that honokiol inhibits the activation of astrocytes rather than microglia and has no effect on CBF in BCAS mice.

### Honokiol Induces OL Maturation Through the Akt/mTOR Pathway

**Fig. 6:**
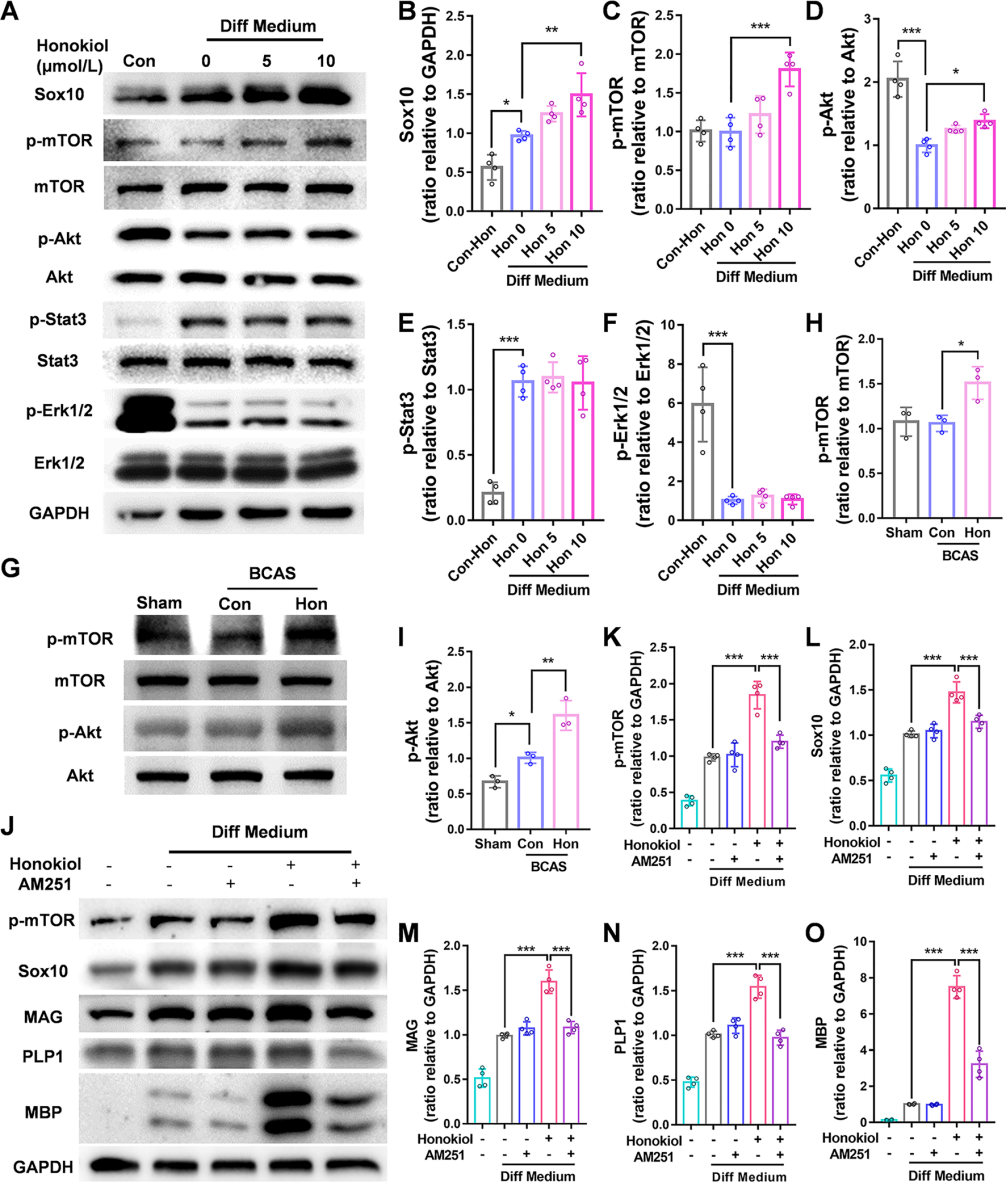
Honokiol up-regulates Sox10 through the CB1/mTOR pathway. **A** Western blots of the expression of Sox10, p-mTOR, mTOR, p-Stat3, Stat3, p-Akt, Akt, p-Erk1/2, and Erk1/2 in primary OPCs treated with 5 or 10 μmol/L honokiol in differentiating medium with GAPDH as a loading control. **B–F** Quantitative analysis of the Western blots as in **A**. **G** Western blots of the expression of p-mTOR, mTOR, p-Akt, and Akt in the corpus callosum 2 months after BCAS. **H, I** Quantitative analysis of the Western blots as in **B**. **J** Western blot of the protein expression of Sox10, p-mTOR, MAG, PLP1, and MBP in primary OPCs cotreated with 10 μmol/L honokiol and/or 10 nmol/L AM251 (a CB1 antagonist) for 24 h with GAPDH as a loading control.** K–O** Quantitative analysis of the Western blots as in **J**. All results are expressed as the mean ± SD. *n* = 4, *P <0.05, **P <0.01, ***P <0.001, NS no statistically significant difference, one-way ANOVA.

To investigate the potential mechanisms by which honokiol modulates OL maturation, we further tested several pathways reported to be affected by honokiol: the Akt/mTOR, Stat3, and ERK pathways. We found that only phosphorylation of Akt and mammalian target of rapamycin (mTOR) was significantly increased after honokiol treatment (Fig. 6A–F). The mTOR has been reported to promote OPC differentiation and myelination *in vivo* [40]. Then we confirmed that phosphorylation of Akt and mTOR was also up-regulated in the honokiol-treated BCAS group (Fig. 6G–I). The above data suggested that honokiol might up-regulate Sox10 by modulating the Akt-dependent mTOR pathway both *in vivo* and *in vitro*. Since honokiol is known to be an agonist of cannabinoid receptor 1 (CB1) [41], we hypothesized that the effect of honokiol on mTOR and Sox10 acted through CB1. Next, the expression of p-mTOR and Sox10 was not enhanced when primary OPCs were exposed to honokiol and AM251, a selective antagonist of CB1 [42] (Fig. 6J–O) for 24 h. AM251 also inhibited the up-regulation of the mature OL proteins MBP, MAG, and PLP1 induced by honokiol in primary OPCs (Fig. 6J–O). Collectively, we showed that honokiol might serve as a CB1 agonist and induce OL maturation by up-regulating Sox10 *via* the Akt/mTOR pathway.

## Discussion

Although the number of patients suffering from WMI with vascular cognitive decline is higher in the aging population, to date there are still no established approaches for this condition [43]. Currently, the BCAS rodent model is commonly applied to mimic WMI and the cognitive deficits caused by cerebral hypoperfusion [44]. Our findings for the first time provided evidence that honokiol can significantly ameliorate cognitive decline by inducing OL maturation in BCAS mice.

A current study has reported that OPCs increase rapidly after chronic cerebral ischemia [45], but most of them fail to differentiate into mature OLs to wrap neuron axons [46]. Recently, modulating OPC differentiation has been regarded as a promising target to attenuate WMI and cognitive impairment after chronic cerebral ischemia [13]. We found that both honokiol and magnolol promote primary OPC differentiation*.* Nevertheless, their effects are not identical, and honokiol was clearly more effective. Although honokiol and magnolol share almost the same chemical structure, some studies have revealed that they also differ to some extent [47]. In a model of glutamatergic and inflammatory pain, honokiol was more selective than magnolol for inhibition of NMDA-induced licking behavioral and thermal hyperalgesia, while magnolol was more potent in blocking CHPG-mediated thermal hyperalgesia [48]. Moreover, honokiol did not significantly induce primary OPC differentiation without T3 treatment. Thus, we conclude that honokiol enhances the T3-potentiated differentiation of OPCs *in vitro*.

The OPCs are generated from the SVZ of the lateral ventricle and dispersed throughout the whole brain, especially the white matter tracts, and then differentiate into mature oligodendrocytes [49, 50]. Thus, we evaluated the integrity of myelin not only in the CC, which is the largest white matter tract in the brain connecting the two hemispheres, but also in the EC and STR, which are also rich in nerve fibers and myelin sheaths [51]. We found that the myelin sheath thickness was reduced and myelin integrity was compromised, but improved by honokiol treatment. in the area of the CC, EC, and STR after BCAS. Since extensive myelin sheath injury leads to cognitive decline, especially working memory [37], we investigated the memory function of BCAS mice by the Y-maze, NOR test, cued FC, and contextual FC test as in previous research [32, 52]. Our results showed that honokiol could significantly ameliorate the dysfunction of working memory (Y-maze and NOR test) and tone-dependent fear memory, rather than context-dependent fear memory, after chronic cerebral ischemia. We suspect that honokiol had limited effects on rescuing contextual fear memory injury due to its strong relationship with hippocampal neuronal function [53].

It is known that OPC differentiation and myelination are orchestrated by various cellular and molecular signals, such as growth factors, extracellular signaling molecules, and neuronal activity [17]. Moreover, the transcriptional factor Sox10 has been reported to be a major determinant in the differentiation of OPCs by up-regulating some critical proteins such as MYRF [54]. CC1 is a mature OL marker that is widely used to label the nuclei of OLs [55], and EdU is a better alternative to BrdU (bromodeoxyuridine) for exploring DNA replication; it also marks the proliferation and regeneration of cells well *in vivo* [56]. Our data showed that honokiol significantly raised the number of newborn OLs (EdU^+^CC1^+^ cells) and total OLs (CC1^+^ cells) in the white matter tract after BCAS, as well as up-regulated Sox10 expression, indicating that honokiol accelerated OL regeneration to enhance remyelination after chronic ischemia. However, we confirmed that honokiol had no effect on OPC proliferation *in vivo*, because there was no change of proliferating OPCs (Ki67^+^NG2^+^ cells) in the SVZ and a decrease of total OPCs (NG2^+^ cell) in the CC after honokiol treatment.

As the current study showed, BCAS mice exhibit a significant decrease in CBF and a deficit of eNOS, which exacerbates WMI, BBB leakage, and inflammatory response, eventually developing into vascular dementia [33]. More and more evidence indicates that microglia and astrocytes contribute to white matter homeostasis. While they are reactive, WMI in chronic cerebral ischemia could be aggravated [35–37]. In addition, it has been reported that Honokiol can reduce the inflammatory response of microglia and astrocytes in the lipopolysaccharide model or ischemic stroke [24, 34]. We demonstrated that honokiol could suppress the activation of astrocytes rather than microglia after experimental cerebral hypoperfusion, with no change in CBF and eNOS expression in the BCAS group. Since astrocytic inflammation has been recognized to exacerbate myelin injury and prevent OL maturation [57], we consider that the inhibition of astrocytic activation by honokiol may also assist in promoting OL regeneration. Our previous research also showed the beneficial role of microglial activation in the BCAS model *via* facilitating the microglial phagocytosis of myelin debris [32]. We believe that, since BCAS has a mechanism distinct from ischemia stroke, the effect of honokiol on microglia involved in the two models may be different.

Honokiol has been identified as a CB1 agonist in a mood disorder model [41]. Relationships between cannabinoids and OLs have been described in other research. CB1 and CB2 are widely expressed in OL lineage cells, and play an essential role in supporting their survival and maturation [58]. Activation of CB1 or CB2 can remarkably accelerate OL differentiation through the Akt/mTOR pathways [59, 60]. The Akt/mTOR pathways are engaged in the proliferation, differentiation, and survival of various cells. Lipid and cholesterol biosynthesis controlled by Akt/mTOR pathways is also involved in OL maturation [61, 62]. We therefore investigated the potential interactions among honokiol, CB1, and Akt/mTOR pathways in the differentiation of primary OPCs. Our data suggested that honokiol can activate CB1, which was confirmed by using a CB1 antagonist AM251, and then promoting OPC differentiation through the Akt/mTOR pathway.

In summary, these findings provide strong evidence that honokiol may be a new strategy for ischemic WMI.

### Supplementary Information

Below is the link to the electronic supplementary material.

Supplementary file1 (PDF 494 kb)
