# Supplementary material for: Compound from Magnolia officinalis Ameliorates White Matter Injury by Promoting Oligodendrocyte Maturation in Chronic Cerebral Ischemia Models
Source: Neurosci Bull. 2023 Jun 9;39(10):1497–511. doi: 10.1007/s12264-023-01068-z (PMC10533772; doi:10.1007/s12264-023-01068-z)
Supplement: Supplementary file 1 — Supplementary file1 (PDF 494 kb) [file 12264_2023_1068_MOESM1_ESM.pdf]

Supplemental Materials

Supplemental Figures

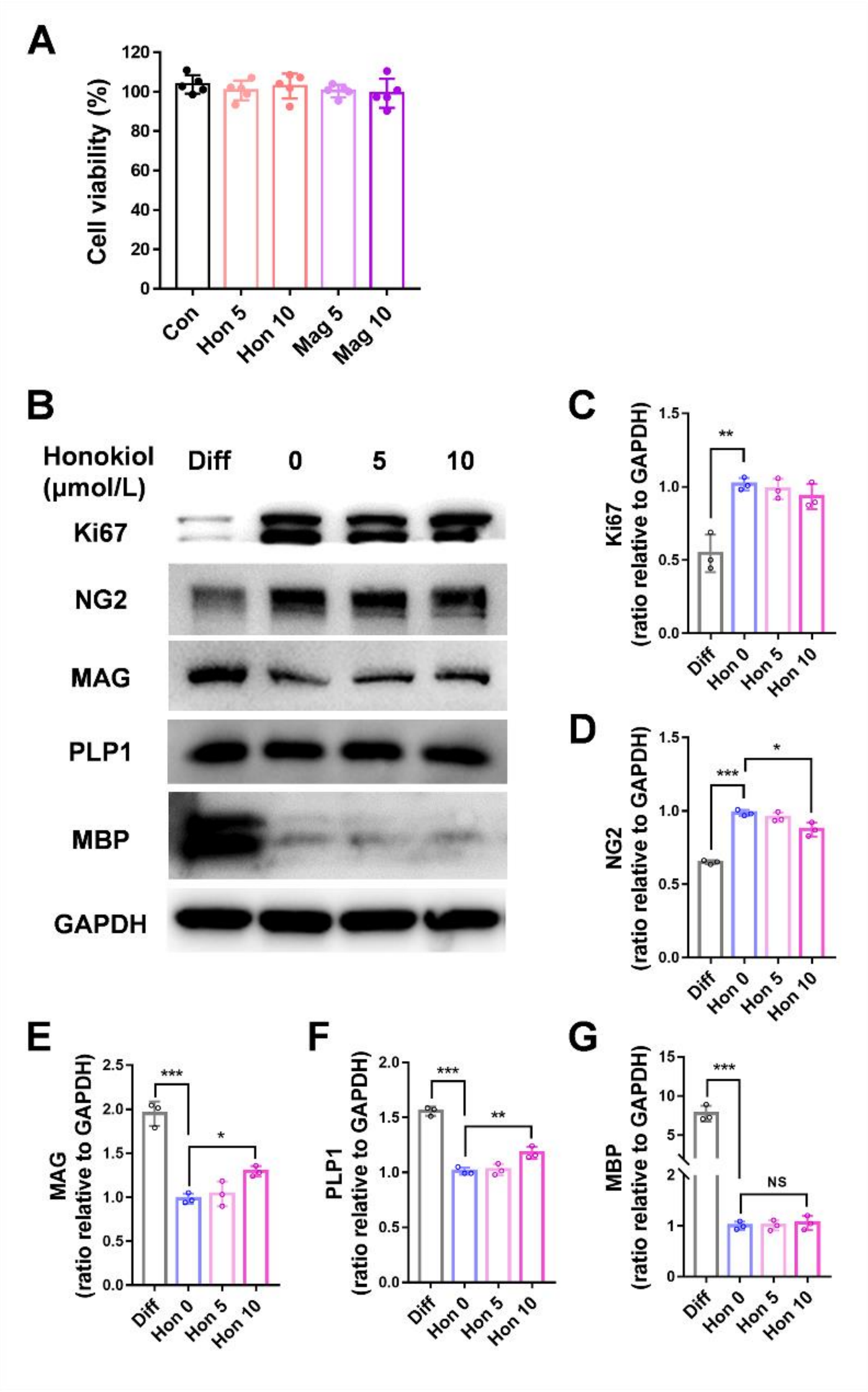

Fig. S1 Effects of honokiol and magnolol on OPC survival, proliferation, and differentiation in proliferating medium. **A** Cell viability of OPCs after treatment with honokiol (5 and 10 μmol/L) and magnolol (5 and 10 μmol/L) for 72 h measured by the CCK-8 test, compared with the control group. **B** Western blots of the expression of Ki67, NG2, MAG, PLP1, and MBP in OPCs treated with honokiol (5 and 10 μmol/L) in proliferating medium with GAPDH as a loading control. **C–G** Quantitative analysis of the Western blots as in **B**. The results are presented as the mean ± SD. $n = 3$, $^*P < 0.05$, $^{**}P < 0.01$, $^{***}P < 0.001$, one-way ANOVA.

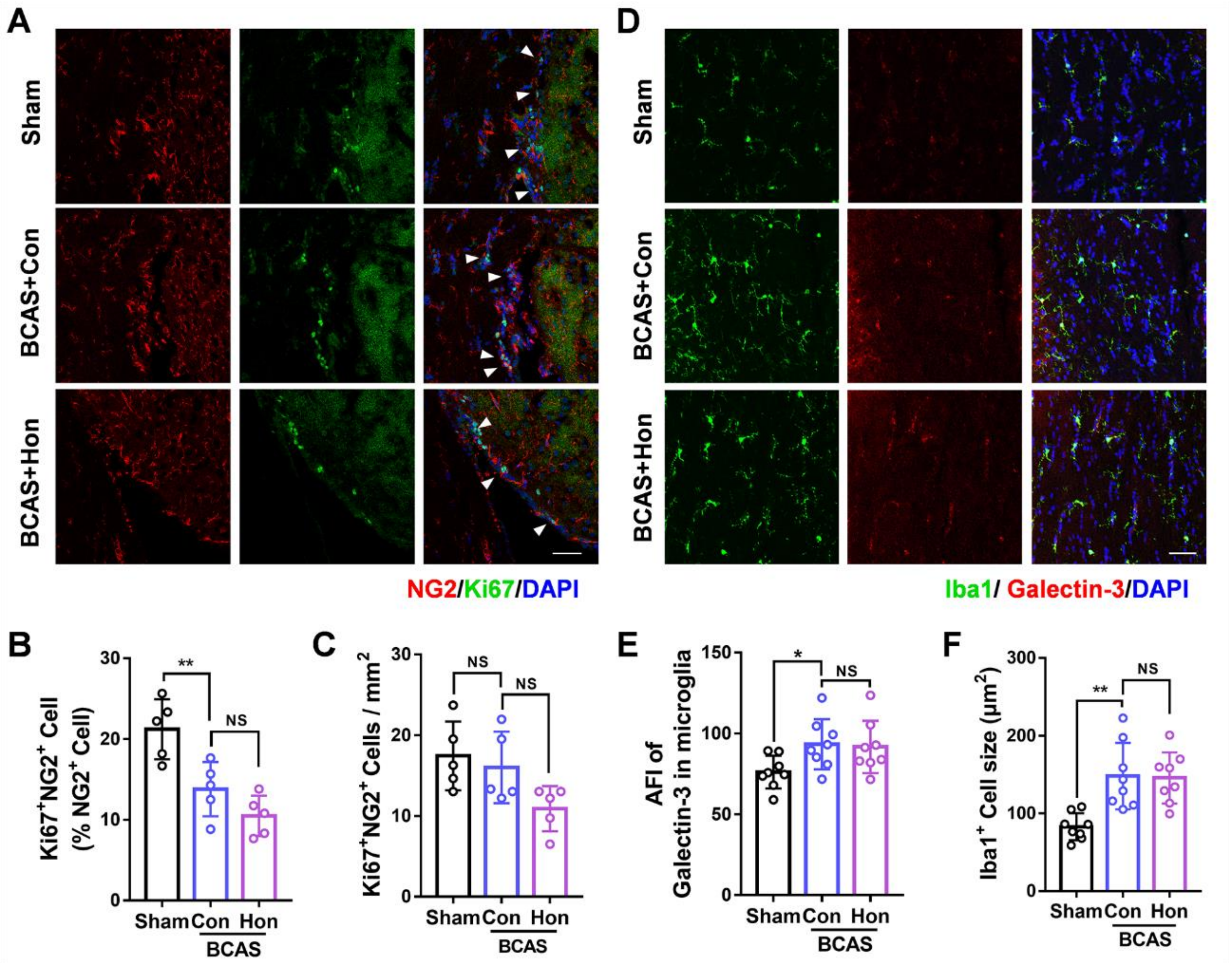

Fig. S2 Effect of honokiol on OPC proliferation and inflammatory activation of microglia in the BCAS model. **A** Representative images of Ki67$^+$ and NG2$^+$ cells (white arrowheads) in the SVZ for each group after BCAS. Scale bar, 50 μm. **B, C** Percentages of Ki67$^+$NG2$^+$ cells in NG2$^+$ cells and number of total Ki67$^+$NG2$^+$ cells per mm^2 as in **A**. **D** Representative images of immunofluorescence staining of galectin-3 (red) and Iba1 (green) in the corpus callosum at 2 months after BCAS. Nuclei are stained with DAPI. Scale bar, 50 μm. **E** Average fluorescence intensity (AFI) of galectin-3 in Iba1$^+$ cells as in **E**. **F** Average size (μm^2) of Iba1$^+$ cells as in (**E**). The results are presented as the mean ± SD. $n = 5$, *P <0.05, **P <0.01, NS no statistically significant difference, one-way ANOVA.

Supplemental Table

Table S1 Details of antibodies

Antibodies	Manufacturer	Catalog number	Application
Rabbit anti-NG2	Millipore	AB5320	WB (1:1000)<br>IF（1:250）
Mouse anti-NG2	Millipore	MAB5384	IF（1:250）
Rabbit anti-Olig2	Millipore	MABN50	IF（1:500）
Rabbit anti-MBP	Abcam	Ab7349	WB (1:1000)<br>IF (1:500)
Rabbit anti-Sox10	Abcam	Ab155279	WB (1:2000)
Goat anti-Iba1	Abcam	AB178846	WB (1:2000)<br>IF (1:500)
Rabbit anti-MAG	Abclone	A9671	WB (1:1000)
Rabbit anti-PLP1	Abclone	A20009	WB (1:1000)
Rabbit anti-Ki67	Abclone	A2094	IF (1:100)
Mouse anti-eNOS	BD Biosciences	610297	WB (1:1000)
Rabbit anti-GAPDH	Bioworld	AP0063	WB (1:5000)
Mouse anti-GFAP	Cell Signaling Technology	3670S	WB (1:2000)<br>IF (1:500)

Rabbit anti-mTOR	Cell Signaling Technology	2972S	WB (1:1000)
Rabbit anti-p-mTOR	Cell Signaling Technology	2971S	WB (1:1000)
Rabbit anti-Akt	Cell Signaling Technology	4685S	WB (1:1000)
Rabbit anti-p-Akt	Cell Signaling Technology	9271S	WB (1:1000)
Rabbit anti-Stat3	Cell Signaling Technology	9139S	WB (1:1000)
Rabbit anti-p-Stat3	Cell Signaling Technology	9145S	WB (1:1000)
Rabbit anti-Erk1/2	Cell Signaling Technology	4695S	WB (1:2000)
Rabbit anti-p-Erk1/2	Cell Signaling Technology	4370S	WB (1:2000)
Rat anti-Galectin-3	BioLegend	125402	IF (1:500)
